# A Cone-Beam Computed Tomography-Based Evaluation of Root Dilaceration in Permanent Premolars: A Retrospective Study

**DOI:** 10.30476/dentjods.2023.98244.2067

**Published:** 2024-06-01

**Authors:** Bahar Asheghi, Safoora Sahebi, Zeinab Rafiee, Maryam Zangooei Booshehri, Afsane Habibi

**Affiliations:** 1 Dept. of Endodontics, School of Dentistry, Shiraz University of Medical Sciences, Shiraz, Iran; 2 Postgraduate Student, Dept. of Endodontics, School of Dentistry, Shiraz University of Medical Sciences, Shiraz, Iran; 3 Oral and Maxillofacial Radiologist, Shiraz University of Medical Sciences, Shiraz, Iran; 4 Dentist, School of Dentistry, Shiraz University of Medical Sciences, Shiraz, Iran

**Keywords:** Cone-beam computed tomography, Dental abnormality, Premolar teeth, Panoramic radiography

## Abstract

**Statement of the Problem:**

As a developmental disorder characterized by an abnormal bend and angle in the longitudinal axis of the tooth root, dilaceration can cause complications in routine dental procedures such as endodontics, orthodontics, and surgical treatments.

**Purpose:**

The purpose of this study was to investigate the prevalence of dilaceration in maxillary and mandibular premolar teeth in a population of Shiraz city based on cone-beam computed tomography (CBCT).

**Materials and Method:**

This is a retrospective cross-sectional study on 927 premolar teeth and 132 CBCT radiographs of patients obtained from four private radiology clinics in Shiraz (Iran). In this study, the presence, location, direction, and severity of dilaceration in premolar roots as well as its relationship with gender were investigated. Chi-square and Fisher tests were used to analyze the data.

**Results:**

The results showed that 17% of the studied 927 teeth had dilaceration. The prevalence of dilaceration was significantly higher in women than in men (20.3% vs. 13.6%, *p*= 0.005). The dilaceration rates were significantly higher in the mandibular first and second premolar teeth (31.6% and 26%, *p*= 0.002) than in the other teeth. In addition, the highest prevalence was in the distal direction with mild severity in the apical third of the root (*p*< 0.001).

**Conclusion:**

According to the results of this study, the prevalence of dilaceration was relatively high in mandibular premolar teeth especially in women.

## Introduction

Dilaceration is considered a dental abnormality related to tooth growth disorder. This disorder can occur in the crown or root of the tooth; it is often seen as a sharp bend or curve [ 1 ].

A successful root canal treatment of permanent teeth requires knowledge and awareness of root anatomy and anomalies. Dilacerations are one of these root anatomy defects and endodontists must pay attention to them when they are planning endodontic treatment, especially in instrumenting the root canal. This will require more radiographic examinations such as Cone-beam computed tomography (CBCT) along with routine clinical examinations.

Dilaceration is sometimes described as an abnormal angle greater than (or equal) to 20 degrees [ 2 ] or greater than or equal to 90 degrees [ 3 - 4 ].

Trauma and damage to deciduous teeth is one of the causes of the occurrence of dilaceration. Injuries such as subluxation and intrusion of deciduous teeth can damage the calcified part of the tooth and result in an abnormal tooth growth in direction and angle [ 5 ]. However, trauma is not considered a principal and definitive factor, especially in posterior teeth. This is because the prevalence of traumatic injuries to primary teeth is reported to be 11-30%, whereas the prevalence of dilaceration in permanent teeth is much lower [ 6 - 7 ]. Hence, several factors should be considered as the causes of dilaceration, especially in posterior teeth. Gender, race, and mechanical factors interfering with tooth growth (such as the presence of cysts, tumors, and extra teeth) could be the causes of this anomaly [ 8 - 11 ]. On the other hand, the ectopic development of teeth and lack of space can probably be the reasons for the high prevalence of dilaceration in third molar teeth [ 4 ].

Root dilacerations are more common than crown dilacerations, especially in first premolar teeth due to idiopathic developmental anomaly. In contrast, crown dilacerations are more common in maxillary and mandibular first incisor teeth and are often associated with a history of trauma to primary teeth [ 12 ].

It has also been reported that apical root dilacerations affecting between 1 and 4.9% of permanent teeth are more common in premolar, incisor, and canine teeth. They are also more common in the maxilla than in the mandible. In general, although dilaceration is observed in both permanent and deciduous tooth systems, it does not belong to a specific permanent tooth [ 4 ].

According to some studies conducted on dental anomalies, dilaceration is the most common developmental anomaly in different populations in both males and females [ 13 - 15 ]. In some other studies, however, dilaceration has been reported as the least prevalent anomaly [ 16 - 17 ]. In most previous studies, conventional radiography and oral Pantomography (OPG) were used to investigate dilacerations. CBCT has recently been accepted as an effective method in evaluating root and canal morphologies and complexities [ 18 - 19 ]. By removing distortion and superimposition, CBCT can give a three-dimensional view of root curvatures in the mesial, distal, buccal, and lingual directions, which cannot be observed efficiently and accurately in two-dimensional radiographs. Moreover, the location, severity, and direction of the dilaceration angle can be seen and measured accurately by using this advanced modality [ 20 - 22 ].

Even though race and geographic location have been reported as influential factors in the prevalence of dental anomalies [ 9 ] and studies in different populations have reported conflicting results, no studies focusing only on dilaceration in the premolar dental group using CBCT have been conducted in Iran.

Since Iran is a vast country, only a few studies have been conducted on the prevalence of dilaceration in its population and most of these studies have employed conventional radiography [ 7 , 14 , 23 ]. Therefore, the present research aimed to investigate the prevalence of dilaceration in mandibular and maxillary premolar teeth by employing CBCT. 

## Materials and Method

This study is a retrospective cross-sectional study on 132 existing CBCT radiographs of patients obtained from four private radiology clinics in Shiraz (Iran) from 2015 to 2022. To avoid unnecessary radiation, the archived CBCTs of the clinics were used. These CBCT stereotypes included 927 premolar teeth.

The CBCTs of premolar teeth in this study, belonging to 479 women and 448 men (18-65 years old), had been taken for different reasons such as placing an implant, jaw fracture, and tumor diagnosis and treatment plan. The Ethics Committee of Shiraz University of Medical Sciences provided the ethical approval (IR.SUMS. DENTAL.REC.1398.33) and consent forms were filled out by all patients.

Of the 927 premolar teeth, 528 samples were first and second premolar teeth in the upper jaw (maxilla) and 399 samples were first and second premolar teeth in the lower jaw (mandible).

The exclusion criteria were impacted teeth, supernumerary teeth, and cleft palates, teeth with external and internal resorption in the root, primary teeth, and permanent teeth with an open apex. It is noteworthy that only teeth with developed roots and complete apices were included in the present research.

After the evaluation of the CBCTs, the variables of this study, including the number of dilacerations in all the premolar teeth, gender, type of tooth (first and second premolars), the tooth position in the jaw (maxilla or mandible), the severity of dilacerations according to the classification of Santana [ 24 ], location in the root (apical, middle or cervical), the direction of dilacerations (lingual, distal, mesial, or buccal), and teeth with S-shaped roots, were recorded and prepared for statistical analysis.

Chohayeb [ 2 ] considered dilaceration as a curvature greater than or equal to 20 degrees on the root surface. In the current research, Chohayeb definition of dilaceration was used.

**Figure 1:**
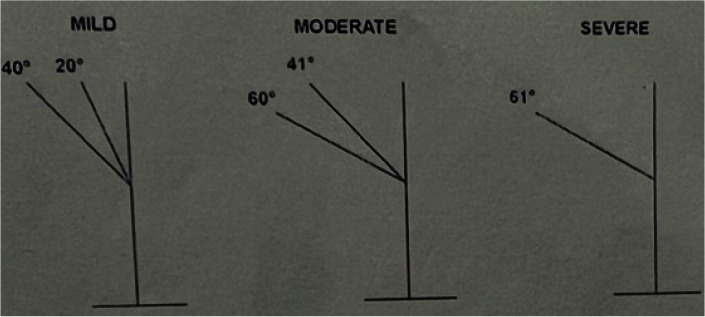
The classification of dilacerations (mild, moderate, or severe), Source: Santana [ 24 ], and Schneider [ 25 ]

**Figure 2:**
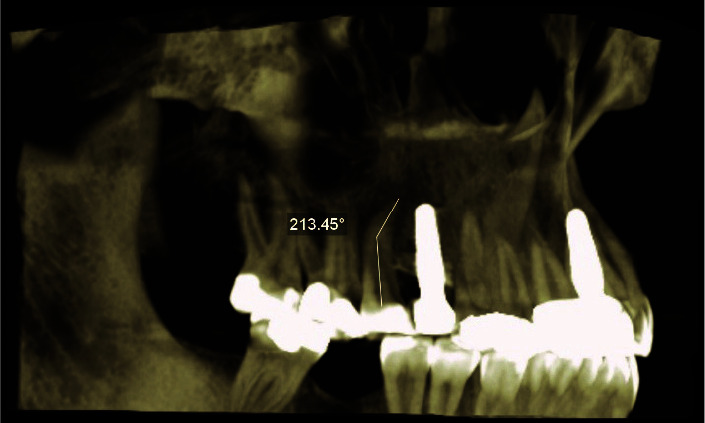
Moderate (41-60֯◌) dilaceration in the right second maxillary premolar in the 3D plane

The severity of dilacerations was based on Santana’s study [ 24 ] in which severity is divided into three groups: mild (20 to 40 degrees), moderate (41 to 60 degrees), and severe (greater than 60 degrees) (Figure 1). In addition, to determine this angle, Schneider’s method [ 25 ] was employed. In this method, one point is placed at the orifice, one at the beginning of the bend, and the last point is placed at the apical foramen. From the first point, a line is drawn parallel to the root and the second and third points are connected with another line. Finally, the size of the angle between the two lines is calculated (Figure 2).

To prepare the CBCT scans, a Planmeca Promax 3D Mid device (Helsinki, Finland) at 90 kVp and 14Ma, with an exposure time of 15 s, and automatically adjusted based on the patient’s body size and weight was employed. A high-definition mode, a maximum field of view of 10*10cm, and a voxel size of 150 μ were used. By employing a magnification tool in the Romexis software, root dilaceration was assessed. The Romexis imaging software (version: 3.8.2) was used to analyze the CBCT images in the sagittal, coronal, and axial sections on a 32-inch monitor in dim light. 

Trained to interpret the images, a calibrated endodontist as well as a senior dental student independently and retrospectively investigated the CBCT images. There was a two-week break between the evaluations. Before the experiments, the investigators assessed sixty other CBCT images. A radiologist assessed the images to achieve a consensus if there was a difference of opinion. To evaluate the reliability of the intra-examiner, a re-assessment was conducted one month after the first session.

### Data analysis

The SPSS software (version: 18.0, SPSS Inc., Chicago IL, USA) was employed for data analysis, while the GraphPad Prism software (version: 8.0) was utilized to create the figures. To assess the qualitative data, Fisher’s exact test and the chi-square statistical test were utilized. The significance level was fixed at *p*<0.05. In the current research, the *p* Value was employed to explore whether root dilaceration statistically depended on jaw type, tooth type, and gender or not. The prevalence of S-shaped roots in the studied teeth was shown as percentages. By employing Cohen’s kappa coefficient, the agreements between the intra- and inter-examiners were computed.

## Results

**Figure 3:**
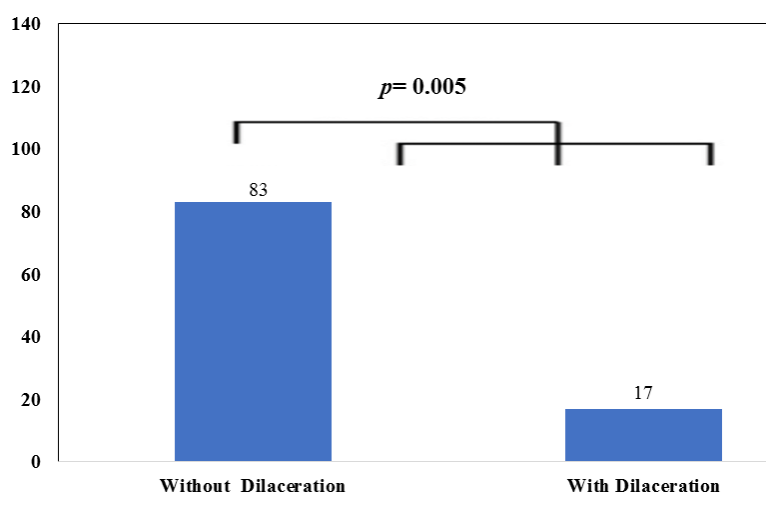
The prevalence of dilaceration in the studied teeth

In the current study, 132 CBCT radiographs were studied to examine the presence of dilacerations in 927 maxillary and mandibular premolar teeth. According to the results, of the 927 studied teeth, 158 teeth (17%) had dilacerations (Figure 3).

Dilaceration in premolar teeth was significantly more prevalent in women (n=97, 20.3%) than in men (n=61, 13.6%) (*p*= 0.005) (Table 1).

**Table 1:** The distribution of dilaceration between the two genders

	Female	Male	Total	*p* Value
Teeth without dilaceration	382 (79.7%)	387 (86.4%)	769 (83%)	*p*= 0.005
Teeth with dilaceration	97 (20.3%)	61 (13.65%)	158 (17%)	
Total	479 (100%)	448 (100%)	927 (100%)	

The results demonstrated that dilaceration was significantly more prevalent in the mandible (n=91, 22.8%) than in the maxilla (n=67, 12.7%) (*p*< 0.001) (Table 2). 

**Table 2:** The distribution of the dilacerated teeth based on the jaws

	Maxilla	Mandible	Total	*p* Value
Teeth without dilaceration	461 (87.3%)	308 (77.2%)	769 (83%)	*p*< 0.001
Teeth with dilaceration	67 (12.7%)	91 (22.8%)	158 (17%)	
Total	528 (100%)	399 (100%)	927 (100%)	

**Figure 4:**
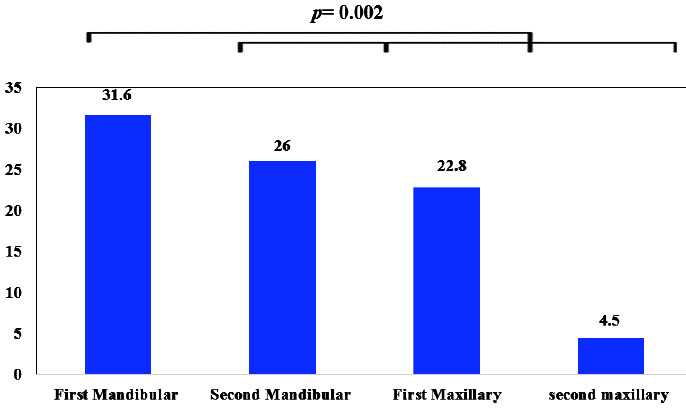
The prevalence of dilaceration in each every studied tooth

It was also shown that the prevalence of dilaceration among the 927 teeth was in the following order: first mandibular (31.6%), second mandibular (26%), first maxillary (22.8%), second maxillary (19.6%) (*p*= 0.002) (Figure 4).

**Figure 5:**
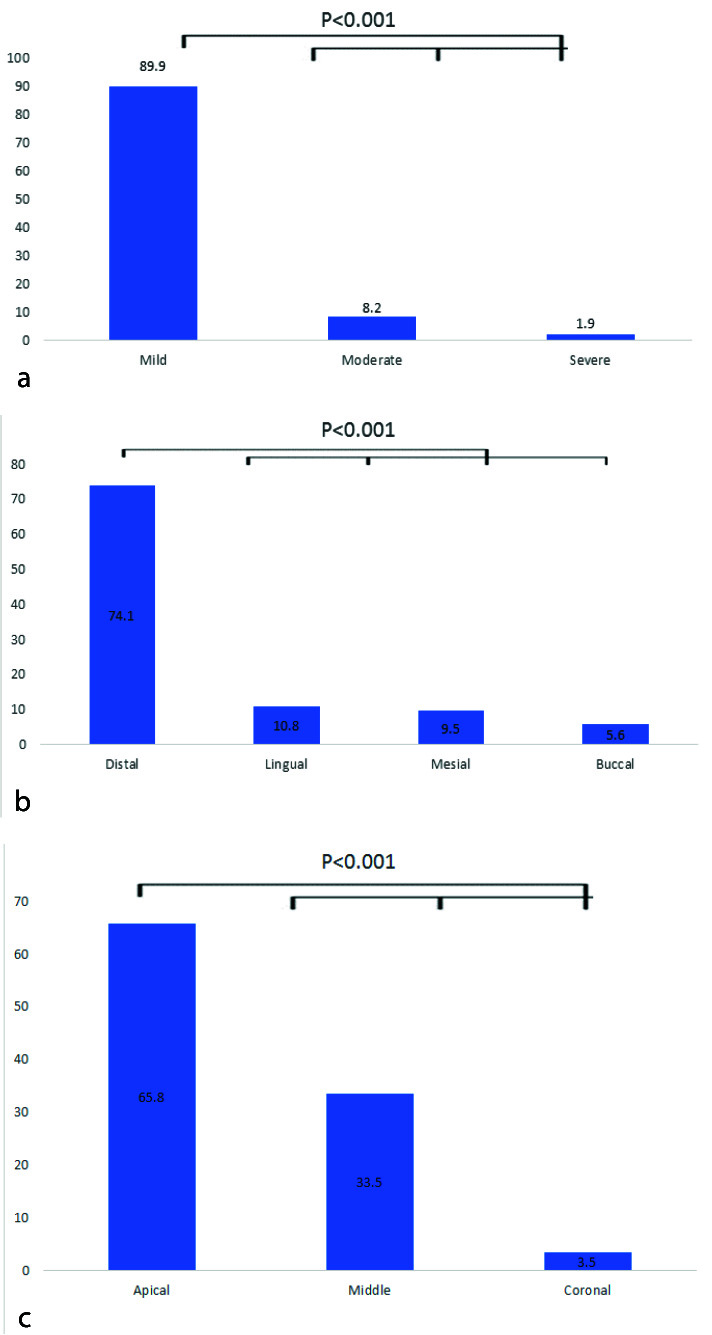
**a:** The severity, **b:** Direction, and **c:** Location of dilacerations in the root length

Mild dilacerations (89.9%) were significantly higher than moderate (8.2%) and severe (1.9%) dilacerations (*p*< 0.001) (Figure 5a).

Generally, dilaceration was most prevalent in the distal direction (74.1%, *p*<0.001) and at the apical one-third (65.8%, *p*< 0.001) (Figure 5b-c). Nonetheless, no S-shaped root was observed in the studied premolar teeth (N=0). 

Cohen’s kappa coefficients for the first and second evaluations were respectively 0.992 and 0.994 with respect to the inter-examiner’s agreement (after the training session). For the intra-examiner’s agreement, the overall Cohen’s kappa coefficient was 0.997. Altogether, this outcome shows that the inter- and intra-examiners had a strong agreement.

## Discussion

Having sufficient knowledge about the anatomy, variations, and developmental disorders of the root is necessary for a successful root treatment. Dilaceration is one of the most important anomalies affecting endodontic treatments [ 26 ]. Therefore, before treating the tooth, it is essential to diagnose the dilacerations.Frequent errors such as ledge, transport, zipping, and broken file are the results of abnormal root canal curvature, which in turn can affect the outcome of endodontic treatment procedures [ 27 ].

This developmental alteration has not been extensively studied and data on its origin, frequency, gender preference, most frequently affected group of teeth and most commonly involved teeth is controversial [ 28 ].

Because it may not be possible to achieve a successful endodontic treatment in severe instances of dilaceration, substitute endodontic treatment methods like vital pulp therapy) direct pulp cap, indirect pulp cap, Cvek pulpotomy, and cervical pulpotomy) can be used [ 2 ].

Some researchers defined a curvature of 90 degrees or more (relative to the longitudinal axis of the tooth) as dilaceration [ 3 , 4 ], while others have considered a curvature of 20 degrees or more as dilaceration in their studies [ 2 ].

Many studies have been conducted to evaluate the prevalence of dilaceration. However, they are largely different regarding their applied methodology. Some studies have used conventional periapical radiography, while others have applied panoramic and periapical radiography. Some other studies have also used extracted teeth. The prevalence of dilaceration was much lower in studies conducted on extracted teeth. The reason is the difficulty of extracting curved teeth, which could be done by sectioning their roots through surgery. Conventional radiographs give information on the root morphology in two dimensions and do not give proper information regarding the third dimension, the buccolingual direction of the root and the exact location of dilaceration [ 23 ].

In the present study, different levels of the roots of mandibular and maxillary premolar teeth were examined using CBCT images. The criteria provided by Chohayeb [ 2 ] as well as Santana’s classification [ 24 ] were also used to determine dilaceration based on the curvature of the roots.

Based on the results of the current research, the prevalence of dilaceration in premolar teeth was 17%. In other studies, the prevalence of dilaceration was reported to be from 0.4% to 4% [ 4 , 7 , 27 , 30 ].

Dilaceration has formerly been demonstrated to be the most prevalent dental anomaly in Iran’s population and includes 15% of all anomaly types [ 14 ]. 

The prevalence of dilaceration in Iran has been reported to be 0.2%, 5.98%, and 1.65% in the studies of Nabavizadeh *et al*. [ 23 ], Sahebi *et al*. [ 22 ], and Kuzekanani [ 7 ], respectively.The reasons for the differences among the reports are the different definitions of dilaceration, the number of samples, different races, and the use of conventional radiography.

According to the results of the present study and the results reported by Asheghi *et al*. [ 20 ] and Sahebi *et al*. [ 22 ], dilaceration was less prevalent in premolar teeth than in posterior teeth and more prevalent than in anterior teeth in the studied population. Since dilaceration has been observed in posterior teeth and according to the findings of Asheghi *et al*. [ 20 ], Sahebi *et al*. [ 22 ], and Miloglu *et al*. [ 31 ], it can be verified that the occurrence of dilaceration may be independent of trauma. 

The evaluation of different articles shows that there is a big difference among different populations regarding the prevalence of dilaceration. New studies have shown that when anatomical malformations are considered in endodontic treatments, new technologies like CBCT can be helpful together with conventional radiographs. CBCT radiographs have been vastly employed in root morphology. CBCT can show the three dimensions of the tooth structure and carefully examine the angles of curvature [ 29 ]. As a result, the current research, CBCT was used to evaluate the dilacerations.

In the current research, dilaceration in premolar teeth was significantly more prevalent present in females than in males (61.4% vs. 38.6%, *p*= 0.009). The results of other studies which examined gender in relation to dilaceration were also consistent with those of the present study [ 20 , 32 - 33 ].

Nevertheless, the results of Miloglu *et al*. [ 31 ] and Gupta *et al*. [ 32 ] did not correlate with those of the present study as they reported that the prevalence of dilaceration was higher in males than in females. Another study showed no gender preference in the prevalence of dilaceration [ 35 ].

In this research, dilaceration was significantly more prevalent in the mandible than in the maxilla. The highest prevalence belonged to the first premolar of the mandible, which is in accordance with the study of Miloglu *et al*. [ 31 ]. However, the prevalence of dilaceration in the maxilla was reported to be higher in the studies of Udoye *et al*. [ 30 ], Colak *et al*. [ 32 ], and Gupta *et al*. [ 33 ]. Other researches did not demonstrate any significant difference regarding the prevalence of dilaceration in the two jaws [ 4 , 36 ]. The reasons are probably the different definitions of dilaceration, the applied methods, and the studied populations.

Based on the results of the current research, mild dilaceration had the highest frequency (89.8%, *p*<00.1). Similarly, the results presented by the studies of Silva *et al*. [ 36 ] and Moreau *et al*. [ 37 ] are in accordance with those of the present study.

The curvature of the root was more prevalent in the distal direction (74.1%) than in the other directions. The results of the current research conform to those of most other studies [ 17 , 20 , 36 , 38 ].

Moreover, in line with the results of other studies [ 4 , 34 ], the current research revealed that dilaceration was significantly more prevalent in the apical third (65.8%, *p*<0.001). 

Due to the vastness of Iran as well as the rather high prevalence of dilaceration in the premolars in the current research, we could not generalize these epidemiological data to the whole country. Hence, it is suggested that more researches with larger sample sizes be conducted in different parts of Iran using CBCT radiography.

## Conclusion

Dilaceration in premolar teeth is relatively common. Since the most common direction after the distal direction is the lingual direction, which is not visible in normal radiographs, CBCT could be considered as an auxiliary method in the diagnosis of dilaceration in these teeth.
